# JNK Inhibition Inhibits Lateral Line Neuromast Hair Cell Development

**DOI:** 10.3389/fncel.2016.00019

**Published:** 2016-02-05

**Authors:** Chengfu Cai, Jinchao Lin, Shaoyang Sun, Yingzi He

**Affiliations:** ^1^Department of Otolaryngology, Affiliated Eye and ENT Hospital of Fudan UniversityShanghai, China; ^2^Department of Otolaryngology—Head and Neck Surgery, The First Affiliated Hospital, Xiamen UniversityXiamen, Fujian, China; ^3^Department of Otolaryngology—Head and Neck Surgery, Quanzhou First Hospital Affiliated to Fujian Medical UniversityQuanzhou, Fujian, China; ^4^Key Laboratory of Metabolism and Molecular Medicine, Ministry of Education, Department of Biochemistry and Molecular Biology, Institute of Medical Sciences, School of Basic Medical Sciences, Fudan UniversityShanghai, China; ^5^Research Center, Affiliated Eye and ENT Hospital of Fudan UniversityShanghai, China; ^6^Key Laboratory of Hearing Medicine, Ministry of Health, Affiliated Eye and ENT Hospital of Fudan UniversityShanghai, China

**Keywords:** JNK, SP600125, hair cell, development, zebrafish

## Abstract

JNK signaling is known to play a role in regulating cell behaviors such as cell cycle progression, cell proliferation, and apoptosis, and recent studies have suggested important roles for JNK signaling in embryonic development. However, the precise function of JNK signaling in hair cell development remains poorly studied. In this study, we used the small molecule JNK inhibitor SP600125 to examine the effect of JNK signaling abrogation on the development of hair cells in the zebrafish lateral line neuromast. Our results showed that SP600125 reduced the numbers of both hair cells and supporting cells in neuromasts during larval development in a dose-dependent manner. Additionally, JNK inhibition strongly inhibited the proliferation of neuromast cells, which likely explains the decrease in the number of differentiated hair cells in inhibitor-treated larvae. Furthermore, western blot and *in situ* analysis showed that JNK inhibition induced cell cycle arrest through induction of *p21* expression. We also showed that SP600125 induced cell death in developing neuromasts as measured by cleaved caspase-3 immunohistochemistry, and this was accompanied with an induction of *p53* gene expression. Together these results indicate that JNK might be an important regulator in the development of hair cells in the lateral line in zebrafish by controlling both cell cycle progression and apoptosis.

## Introduction

The zebrafish has become an attractive model organism for studying the molecular and cellular basis of sensory organ morphogenesis. Zebrafish embryos are transparent, and the lateral line system comprises a series of sensory organs, called neuromasts, that are located on the surface of the head and along the body in species-specific patterns. Lateral line neuromasts house hair cells and supporting cells, and hair cells can be readily observed and accessed due to their external location. Hair cells of the zebrafish lateral line exhibit a morphology and function similar to mammalian inner ear hair cells (Raible and Kruse, 2000; Nicolson, 2005), and they have been used as a powerful model for investigating hair cell development and identifying new candidate molecules and pathways that are required for hair cell development (Metcalfe et al., 1985; Riley, 2003; He et al., 2013, 2014; Loh et al., 2014; Thomas et al., 2015). The mitogen activated protein kinase (MAPK) family has been shown to play important roles in regulating many *developmental* processes, including cellular growth, proliferation, differentiation, and apoptosis (Seger and Krebs, 1995; Pearson et al., 2001). The MAPK family is conserved, and three MAPK signaling pathways have been identified: extracellular-signal-regulated kinase (ERK), p38 mitogen-activated protein kinase (p38), and c-Jun N-terminal kinase (JNK; Hanks et al., 1988; Gupta et al., 1996). The JNK subgroup contains three major isoforms in vertebrates that are denoted as JNK1, JNK2, and JNK3 (Kallunki et al., 1994; Gupta et al., 1996; Yoshida et al., 2001; Weston and Davis, 2007). It is well known that the JNK signaling pathway interacts with a variety of other signaling pathways and is activated by stress stimuli or growth signals to execute its functions in cell differentiation, proliferation, apoptosis, inflammatory responses, and nervous system development (Han and Ulevitch, 1999; Davis, 2000; Lin, 2003; Weston and Davis, 2007). Depletion of both *jnk1* and *jnk2* in mice is embryonic lethal due to severe dysregulation of apoptosis in the brain, and this suggests that *jnk1* and *jnk2* are critical in regulating the differentiation and survival of neuronal cells in the nervous system (Kuan et al., 1999; Sabapathy et al., 1999). Targeted disruption of the *jnk3* gene causes the mice to be resistant to glutamate excitotoxicity, but not disruption of the *jnk1* or *jnk2* genes, indicating a specific role of this gene in stress-induced neuronal apoptosis (Yang et al., 1997). Owing to the importance of JNK signaling, studies involving this pathway have been extensive. It has been reported that JNK signal pathway is related to many physiological and pathological processes, such as neuron sprouting (Eminel et al., 2008), tubulin dynamics in migrating neurons (Kawauchi et al., 2003), and progression of cancer (Moon et al., 2008) and numerous other diseases (Salh, 2007; Mehan et al., 2011; Davies and Tournier, 2012).

SP600125 is a synthetic polyaromatic chemical that is widely used as a selective inhibitor of JNK signaling in biochemical studies (Bennett et al., 2001; Han et al., 2001). Treatment with SP600125 reduces the number of mouse embryonic stem cell colonies in culture and inhibits their proliferation by arresting the cell cycle at the G_2_/M phase (Zhou et al., 2013). Recent studies have indicated the physiological roles of JNK signaling in embryogenesis and organogenesis. For example, developmental studies demonstrate that there are distinct expression patterns of JNK family proteins at different embryonic developmental stages and during organogenesis in zebrafish. Reduction of JNK1 by RNA interference results in several defects and malformations of zebrafish embryos. Chemical inhibition of JNK with SP600125 results in high mortality and severe organ abnormalities during embryonic development in zebrafish similar to that caused by knockdown of JNK1 mRNA. In the ovary, pharmacological inhibition of JNK with SP600125 inhibits ovarian differentiation and development in zebrafish during early ontogenetic stages (Xiao et al., 2013). The study by Xie and colleagues reported that the effects of SP600125 on development appear to be multifaceted. In mouse pre-implantation embryonic development, administration of SP600125 decreased the rate of development if embryos were cultured in suboptimal media (Ham's F10), while the rate of development increased when they were in optimal media (Xie et al., 2006). These data also suggest that decreased progression into S phase and increased apoptosis account for the slow increase in cell number in suboptimal media. Previous studies have demonstrated an important role for JNKs in the correct development of the nervous system (Kuan et al., 1999; Shoichet et al., 2006) and in the developing and adult brain, and abrogation of JNK signaling alters neuronal pathfinding, migration, and axodendritic architecture and synaptic function (Coffey, 2014). However, the role of JNK signaling in hair cell development is, so far, not well understood.

The purpose of the current study was to investigate the function of JNK in hair cell development in the zebrafish lateral line. We hypothesized that inhibiting JNK would attenuate the differentiation of hair cells. We investigated the role of JNK using the SP600125 inhibitor in zebrafish larvae. To assess the development of neuromast hair cells, we took advantage of the (*Brn3c*:mGFP) transgenic zebrafish embryo that expresses green fluorescent protein (GFP) in the hair cells of the lateral line neuromasts (Xiao et al., 2005). Our data showed that pharmacologic inhibition of JNK effectively reduced the numbers of hair cells and supporting cells in developing neuromasts, and the effect of SP600125 was dose dependent. We provide evidence that inhibition of JNK significantly inhibited proliferation of the progenitor cell population and induced increased *p53* and *p21* levels during hair cell development. In addition, SP600125 at higher concentration caused significant cell death in lateral line neuromasts. Overall, our study demonstrates that JNK is required for the development of hair cells and that inhibition of JNK directly inhibits cell proliferation and induces cell cycle arrest and apoptosis during the course of hair cell development in zebrafish lateral line neuromasts.

## Materials and methods

### Zebrafish embryos and drug administration

All zebrafish animal experiments were performed following the institutional guidelines approved by the Institutional Animal Care and Use Committee of Fudan University, Shanghai. The ages of zebrafish larvae are described as days post fertilization (dpf). SP600125 was dissolved in dimethyl sulfoxide (DMSO, Sigma Aldrich, St Louis, MO, USA) at a stock concentration of 50 mM and further diluted to the desired concentrations in fresh egg water. SP600125 treatment commenced at 3 dpf, and larvae were separated into four groups and given SP600125 at 0 (control), 5, 10, or 15 μM. Treatment was conducted for 2 days with daily water changes followed by several washes in fresh egg water. The larval zebrafish were then fixed with 4% paraformaldehyde (PFA) in phosphate buffered saline (PBS) at 4°C until further processing.

### Cell proliferation and analysis

Proliferating cells in the lateral line neuromasts were labeled by adding 10 mM 5-bromo-2-deoxyuridine (BrdU; Sigma Aldrich) to the fresh egg water for 2 days at 28.5°C. Larvae were then fixed with 4% PFA overnight at 4°C, and BrdU incorporation was detected by fluorescent immunostaining. The fixed larvae were washed three times in PBS containing 0.5% Triton X-100 (PBT-2) and placed in 2 N HCl for 0.5 h at 37°C. Larvae were blocked in 10% normal goat serum for 1 h at room temperature and incubated with the monoclonal primary anti-BrdU antibody (1:200 dilution; Santa Cruz, Dallas, TX, USA. Cat. no. sc-32323) overnight at 4°C. The next day, larvae were washed three times for 10 min each with PBT-2 and then incubated with the secondary antibody for 1 h at 37°C. Fluorescently labeled larvae were imaged with a Leica confocal fluorescence microscope (TCS SP5; Leica, Wetzlar, Germany).

### Immunohistochemistry

For immunohistochemistry analysis, larvae were fixed with 4% PFA and were permeabilized with PBT-2 for 30 min followed by incubation in blocking solution for 1 h. Primary antibodies were then added overnight at 4°C with rocking. The following antibodies were used as primary antibodies: anti-GFP (1:1000 dilution; Abcam, Cambridge, UK), anti-Sox2 (1:200 dilution; Abcam), and anti-cleaved caspase-3 (1:500 dilution; Cell Signaling Technology Inc., Danvers, MA, USA). After three washes of 20 min, Alexa Fluor 488–, 594–, and/or Alexa Fluor 647–conjugated secondary antibodies (Jackson ImmunoResearch Laboratories, West Grove, PA, USA) were added at a dilution of 1:500 in blocking solution and incubated overnight at 4°C with rocking. Nuclei were labeled with 4,6-diamidino-2-phenylindole (DAPI; 1:800 dilution; Invitrogen, Carlsbad, CA, USA) for 20 min at room temperature. For image collection, Z-sections were taken at 1 μm intervals through the depth of the neuromast. For analyses, maximum intensity projections were generated. Images were processed using Photoshop software (Adobe). Cell counts were performed at the time of imaging by viewing the images using a Nikon Eclipse Ni Fluorescence Microscope (Nikon Instruments) using a 40X objective. Double-labeled cells in neuromasts were counted on a confocal microscope, using a 63X objective (TCS SP5; Leica, Wetzlar, Germany). BrdU+ cells having a shape identical to that of a hair cell or supporting cell and corresponding to the exact location of a neuromast were counted.

### FM1-43FX labeling

The vital dye FM1-43FX (Molecular Probes, Eugene, OR, USA)—which enters mature hair cells through mechanotransduction-dependent activity—was applied at a concentration of 3μM to live 5 dpf larvae for 45 s in the dark. After quickly rinsing three times with fresh water, the larvae were anesthetized in 0.02% MS-222 and fixed with 4% PFA in PBS for 2 h at room temperature or overnight at 4°C.

### Western blot analysis

Total protein was isolated from whole larvae at 5 dpf using AllPrep DNA/RNA/Protein Mini Kit (QIAGEN, Hilden, Germany) according to the manufacturer's instructions. Protein concentrations were measured using a BCA protein kit (Thermo Fisher Scientific, Rockford, IL), and proteins were separated on SDS-polyacrylamide gels and transferred onto PVDF membranes (Immobilon-P; Millipore, Bedford, MA, USA). The membranes were blocked with 5% non-fat dried milk in TBST [50 mM Tris-HCl (pH 7.4), 150 mM NaCl, and 0.1% Tween-20] for 1 h at room temperature and then blotted overnight with primary antibodies at 4°C. The following antibodies were used as primary antibodies: anti-cleaved caspase-3 (1:1000 dilution; Cell Signaling Technology Inc.), anti-p21 (1:500 dilution; Santa Cruz Biotechnology, Inc.), and anti-p53 (1:1000 dilution; Abcam); anti-JNK (1:1000 dilution; Abcam); anti- p-JNK (1:500 dilution; Santa Cruz Biotechnology, Inc.)

### Whole-mount *In situ* hybridization

The probes used in *in situ* hybridization (*jnk1, p21*, and *p53*) were amplified by PCR from zebrafish embryo cDNA using the following primers and cloned into the pGEM-T Easy Vector (Promega, cat. no. A1360): *jnk1* forward: 5′-agtgtgttgtttcctggcac-3′; *jnk1* reverse: 5′-actgctgtcggtgtctgag-3′; *p21* forward: 5′-acaagcggatcctacgttca-3′; *p21* reverse: 5′-ctacgagacgaatgcagctc-3′; *p53* forward: 5′-tcttttgaggtgcgtgtgtg-3′; *p53* reverse: 5′-acatgtatcgcagttcccca-3′. Digoxigenin-labeled antisense RNA probes were generated by *in vitro* transcription using T7 or SP6 RNA polymerase (Promega). Regular whole-mount *in situ* hybridization of zebrafish embryos was performed as previously described (Thisse and Thisse, 2008). Briefly, the embryos were depigmented with 1-phenyl-2-thiourea (PTU, Sigma-Aldrich, cat. no. P7629), euthanized in MS-222, and fixed overnight with 4% PFA at 4°C. The fixed embryos were washed in PBS with 0.1% Tween-20 (PBST) and placed in 100% methanol at –20°C for dehydration. Prior to use, they were rehydrated in a graded methanol series and washed three times for 5 min with PBST. To permeabilize the embryos, proteinase K (10 μg/mL in PBST) was added for 50 min and the embryos were refixed in 4% PFA for 20 min. After washing in PBST, the embryos were pre-hybridized at 65°C for ≥2 h in hybridization buffer. For hybridization, the labeled probes were added to the hybridization buffer at 65°C overnight. After washing for 15 min with 75%, 50%, and 25% hybridization buffer and 2X SSCT (20X SSC, Life technologies, AM9770; 0.1% Tween-20) and for 30 min twice in 0.2X SSC at 65°C, embryos were blocked for at least 2 h at 4°C in blocking buffer (Roche cat. no._11096176001) and were incubated with pre-absorbed sheep anti-digoxigenin-AP Fab fragments (Roche cat. no. 11093274910) at a 1:4000 dilution in blocking buffer overnight at 4°C. The next day, the embryos were washed 4 × 30 min with 2 mg/mL BSA in PBST and 3 × 5 min in staining buffer (100 mM Tris (pH 9.5), 100 mM NaCl, and 0.1% Tween-20). Afterwards, the embryos were stained with BM purple AP substrate (Roche cat. no. 11 442074001) in the dark. Finally, the color reaction was stopped by adding PBST, and the embryos were observed under a bright field microscope (Nikon Instruments).

### Tunel staining

For TUNEL (Terminal deoxynucleotidyl transferase-mediated dUTP nick end labeling) assays, 5 dpf larvae were incubated in 0.1 M glycine/PBS solution for 10 min and then rinsed with PBT-2 three times for 10 min each. The larvae were then processed using the *In situ* Cell Death Detection Kit (Roche, Nutlet, NJ, USA; cat. no. 11684795910) following the directions supplied by the manufacturer.

### Cell counts and statistical analysis

Cells in the first four lateral line neuromasts (L1–L4) were counted. Prior to analysis, all data were first examined for normality and homogeneity of variances by the Shapiro–Wilk test and Levene's test, respectively. For statistical comparisons, differences among groups were compared using one-way ANOVA, and differences between groups were compared using an unpaired *t*-test (two-tail; see figure legends for details). Data were analyzed using SigmaPlot (version 12.0 for Windows; Systat Software Inc., CA, USA). All data are presented as the mean ± SD. A *p* < 0.05 was considered statistically significant.

## Results

### SP600125 treatment affects the development of hair cells and supporting cells in the lateral line

To address the question whether the JNK pathway is involved in the hair cell development of zebrafish, we analyzed the expression pattern of the *jnk1* gene. Whole-mount *in situ* analysis demonstrates that *jnk1* is highly expressed in the neuromasts of zebrafish larvae at 5 dpf (Supplemental Figure 1A). SP600125, an anthrapyrazolone inhibitor of JNK catalytic activity, has been used to inhibit JNK with a high specificity and is widely used for assessing the complex roles of JNK in regulating biological processes (Bennett et al., 2001). To investigate the effects of SP600125 on JNK phosphorylation during hair cell development, zebrafish larvae at 3 dpf were treated with SP600125 for 2 days followed by western blot analysis of the levels of JNK phosphorylation. JNK activation was observed in untreated control larvae at 5 dpf, but SP600125 significantly decreased JNK phosphorylation when compared with 5 dpf control larvae (Supplemental Figure 1B).

To investigate the effects of SP600125 on lateral line hair cell development in zebrafish, we incubated zebrafish larvae in varying concentrations of SP600125 from 3 to 5 dpf, the time during which most of the hair cells are formed and become functional (Raible and Kruse, 2000; Harris et al., 2003). We first assessed the impact of SP6000125 on the development of zebrafish with bright field microscopy. No global defects were observed between the embryos treated with SP6000125 and the control embryos, and there were only a few apparent differences in development between the embryos treated with 15 μM SP6000125 and control embryos. Within these samples, pericardium edema and reduced body length were the obvious external defects in embryos treated with 15 μM SP600125 (Supplemental Figure 2).

**Figure 1:**
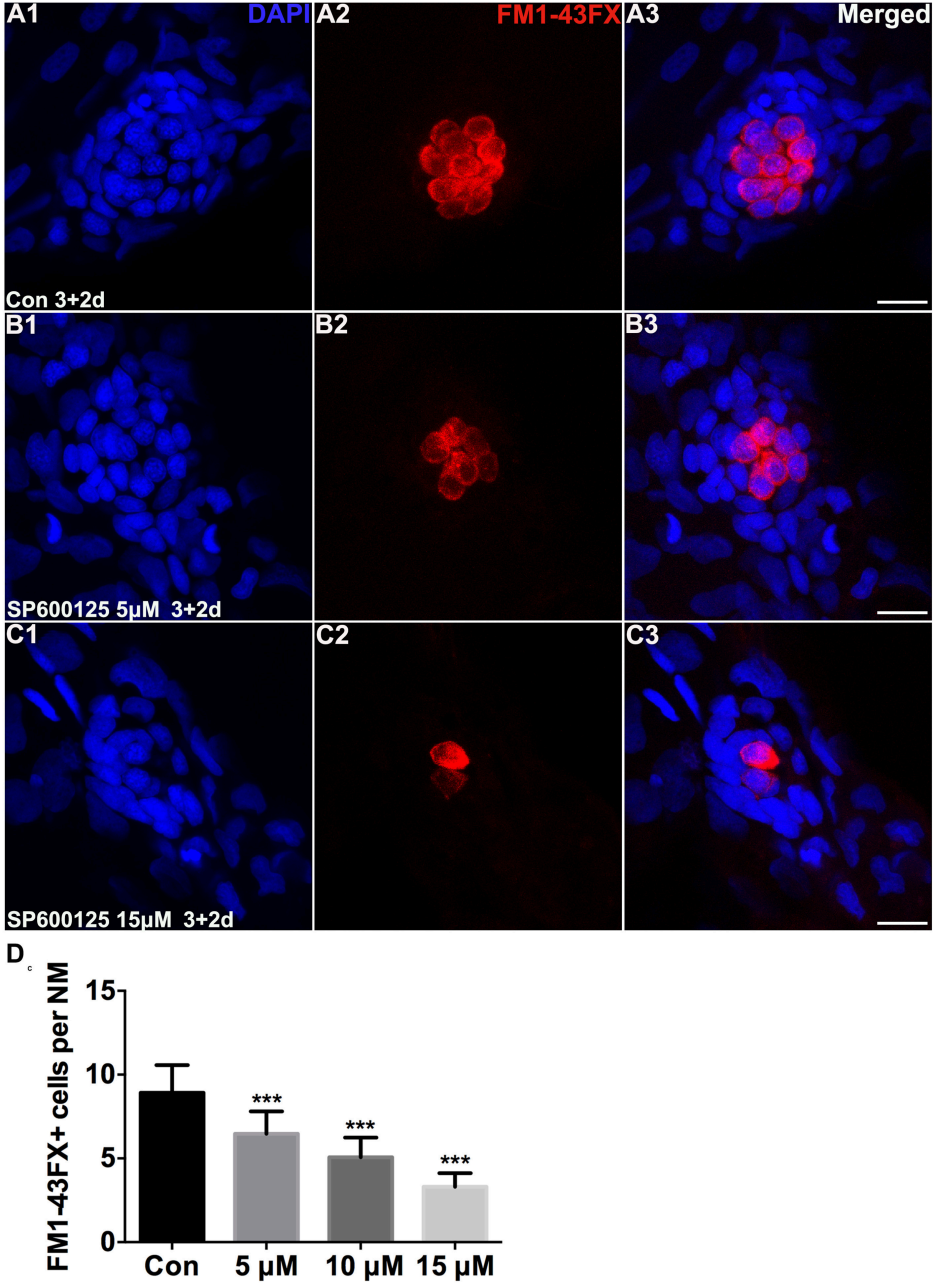
**SP600125 reduced the number of FM1−43FX+ cells**. **(A–C)** We treated 3 dpf zebrafish with or without SP600125 for 2 days and subsequently imaged FM1-43FX+ cells (red). Higher magnification of hair cells of the neuromast taken from z-stacks show that **(A)** hair cells in untreated controls and **(B,C)** SP600125-treated animals had no observable morphological differences though there were fewer hair cells in the SP600125-treated animals. Nuclei are stained with DAPI and scale bars = 10 μm. **(D)** The average number of FM1−43FX+ cells per neuromast (NM) in larvae treated with or without SP600125 for 2 days. The first 4 neuromasts along the body, L1–L4, were recorded on one side of each fish. *n* = 26 neuromasts in control, *n* = 28 in 5 μM SP600125-treated neuromasts, *n* = 40 in 10 μM SP600125-treated neuromasts, and *n* = 28 in 15 μM SP600125-treated neuromasts. One-way ANOVA; FM1−43FX+ cells: F3, 118 = 96.18, *p* < 0.001. Bars are mean ± SD. ^***^p < 0.001, highly significant difference when compared to control larvae.

**Figure 2:**
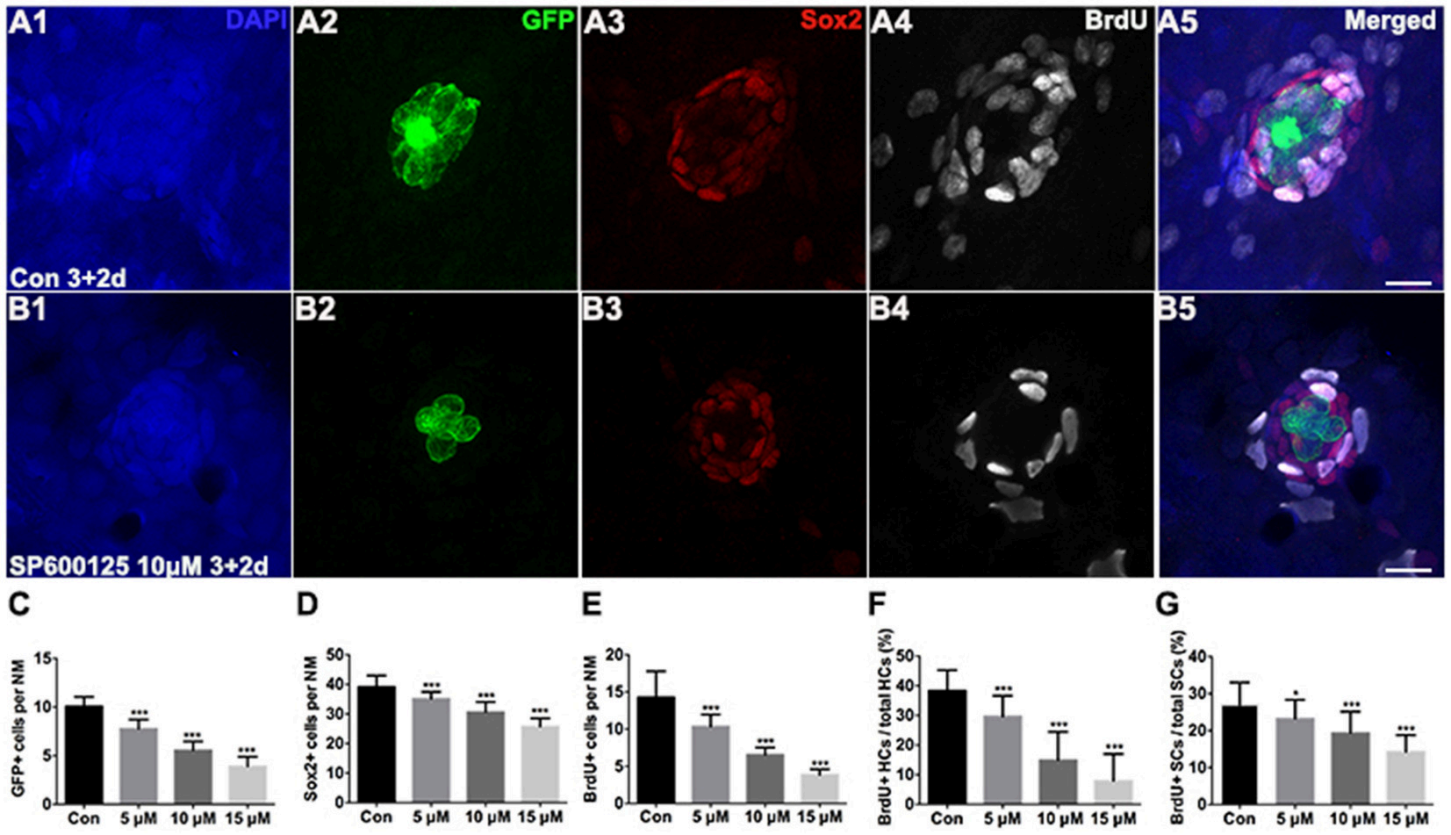
**Detection of neuromast hair cells and supporting cells in 5 dpf larvae**. **(A,B)** Confocal images of neuromasts from a 5 dpf control larva and 5 dpf larva treated with 10 μM SP600125. The neuromast hair cells in the transgenic line *Brn3c:*mGFP were detected by GFP visualization (green), supporting cells were detected by Sox2 (red), and proliferating cells were detected by BrdU (white). Higher magnification of hair cells and supporting cells of the neuromast taken from z-stacks show that hair cells and supporting cells in untreated controls and SP600125-treated animals had no observable morphological differences though there were fewer GFP-positive and Sox2-positive cells in the neuromasts of larvae treated with SP600125. The number of BrdU-labeled cells is much larger in control than in SP600125-treated larvae. Scale bar = 10 μm. **(C,D)** Quantification of hair cells and supporting cells in the neuromast (NM) for each experimental condition. **(E–G)** Quantification of replicating cells in the neuromasts for each experimental condition. SP600125 treatment decreased the numbers of BrdU-positive cells, the ratio of BrdU-positive hair cells, and the ratio of BrdU-positive supporting cells in neuromasts. The first four neuromasts along the body, L1–L4, were recorded on one side of each fish [one-way ANOVA; GFP+ cells: *F*_(3, 112)_ = 237.5, *p* < 0.001; Sox2+ cells: F_(3, 112)_ = 102.5, *p* < 0.001; BrdU+ cells: F_(3, 112)_ = 134, *p* < 0.001; BrdU+ HCs: *F*_(3, 112)_ = 89.7, *p* < 0.001; BrdU+ SCs: *F*_(3, 111)_ = 32.08, *p* < 0.001]. Bars are mean ± SD. *n* = 36 neuromasts in control, *n* = 28 in 5 μM SP600125-treated neuromasts, *n* = 24 in 10 μM SP600125-treated neuromasts, and *n* = 28 in 15 μM SP600125-treated neuromasts. ^*^p < 0.05, significant difference when compared to control larvae; ^***^p < 0.001, highly significant difference when compared to control larvae.

To test the functionality of the differentiated hair cells, we stained 5 dpf larvae with the vital dye FM1-43FX, which is a marker of functional mechanotransduction channels in hair cells (Seiler and Nicolson, 1999). We found that the hair cells of SP600125-treated larvae showed overall normal morphology; however, there was a significant reduction in the total number of FM1-43FX-positive hair cells (Figure 1). We also used transgenic (*Brn3c*:mGFP) zebrafish that express GFP in differentiated hair cells (Figures 2A2,B2) to further quantify changes in the numbers of hair cells. Larvae in the control groups harbored 10.08 ± 0.97 GFP-positive hair cells per neuromast (*n* = 36 neuromasts), while the larvae treated with 5 μM SP600125 harbored 7.75 ± 0.97 GFP-positive hair cells (*n* = 28 neuromasts; *p* < 0.001). As shown in Figure 2, with an increasing concentration of SP600125 (10 and 15 μM) the numbers of GFP-positive hair cells were reduced even further (Figure 2C; SP600125 10 μM, 5.54 ± 0.93 GFP-positive cells, *n* = 24 neuromasts, *p* < 0.001 vs. control; SP600125 15 μM, 3.9 ± 1.0 GFP-positive cells, *n* = 28 neuromasts, *p* < 0.001 vs. control). The experiment was repeated three times with consistent results (Supplemental Figure 3). These results confirmed the reduced FM1-43FX-positive hair cells observed in the SP600125-treated group and showed that normal hair cell development is severely impaired in the presence of JNK inhibitor. To test the contribution of ongoing JNK phosphorylation to hair cell differentiation, SP600125 was removed from some larvae at the 5 dpf time point. There was a significant decrease in the number of FM 1-43FX-positive hair cells in the zebrafish larvae at 3 dpf treated for 4 days with SP600125 when comparing the controls (*p* < 0.001) and the inhibitor washout experiment group (*p* < 0.001; Supplemental Figure 4). These washout experiments demonstrated that changes brought about by SP600125 were reversible, and hair cell differentiation resumed upon removal of the inhibitor. We next identified the effects of SP6000125 on supporting cell development by Sox2 immunohistochemistry (Figures 2A3,B3). We quantitatively assessed the Sox2-labeled cells in the neuromasts after treatment with different concentrations of SP600125 and observed a dose-dependent reduction in the number of stained supporting cells (Figure 2D; *p* < 0.001). These results suggest that JNK is involved in neuromast development.

### Impact of SP600125 on neuromast cell proliferation

Because there was a reduction in the number of neuromast hair cells, and because JNK has been shown to be involved in regulating cell proliferation and apoptosis, we hypothesized that SP600125 treatment caused a reduction in cell proliferation, an increase in cell death, or a combination of both. To test the effect of SP600125 on cell proliferation in zebrafish lateral line neuromasts, we incubated 3 dpf larvae with both 10 mM BrdU and SP600125 for 2 days (Figures 2A4,B4). The larvae were fixed at 5 dpf and processed for BrdU immunohistochemistry. SP600125-treated larvae exhibited significantly fewer BrdU-positive cells compared to controls (Supplemental Figures 5A,B). The level of neuromast cell proliferation was determined by counting the number of BrdU-positive cells in the lateral line neuromasts. In control larvae, there were 14.28 ± 3.5 BrdU-positive cells in neuromasts between 3 dpf and 5 dpf. SP600125 treatment caused a significant reduction in the number of BrdU-labeled cells in a dose-dependent manner (Figure 2E; *p* < 0.001).

To distinguish the newly differentiated hair cells from cell proliferation, we quantified the ratio of GFP and BrdU double-labeled cells to GFP-labeled hair cells in neuromasts of 5 dpf larvae (Figure 2F). In control fish, a considerable number of GFP-expressing cells were co-labeled with BrdU, while in SP600125-treated neuromasts the BrdU incorporation was mainly detected in the periphery of the neuromast and there was very little overlap of signals. The ratios of GFP and BrdU double-labeled cells to GFP-labeled cells in neuromasts in SP600125-treated larvae at concentrations >5 μM were significantly lower compared with the untreated larvae (Figure 2F; *p* < 0.001). Because the supporting cells serve as the major source of newly differentiated hair cells within the neuromast, we counted the cells that were double labeled with anti-Sox2 and anti-BrdU antibodies and calculated the ratio of double-labeled cells to the Sox2-labeled cells. The percentage of double-labeled cells was significantly reduced by SP600125 treatment for 2 days starting at 3 dpf, and the effect was dose dependent (Figure 2G*; p* < 0.05, 0.001). These findings suggest that SP600125 significantly decreased the BrdU-labeled cells indicating that there are fewer cells progressing into S-phase of the cell cycle in the neuromast.

### SP600125 administration induces apoptosis and cell cycle arrest in zebrafish neuromasts

**Figure 3:**
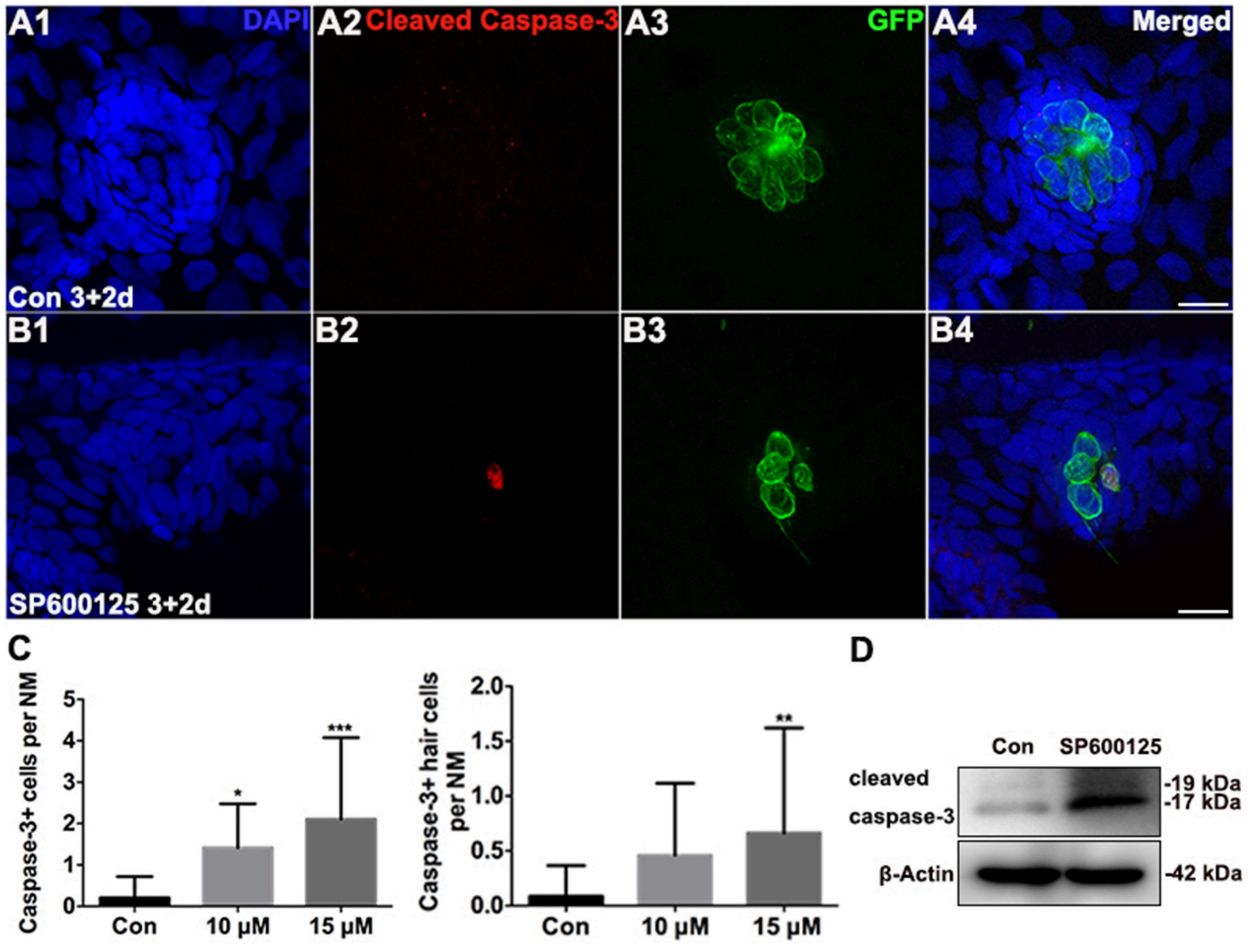
**Effects of SP600125 on apoptosis**. **(A–B)** Cleaved caspase-3 staining in the neuromast from a 5 dpf control larva **(A)** and 15 μM SP600125-treated 5 dpf larva **(B)**. Scale bar = 10 μm. **(C)** SP600125 treatment increased the numbers of cleaved caspase−3−positive cells and cleaved caspase−3−positive hair cells [one-way ANOVA; Caspase−3+ cells: *F*_(2, 101)_ = 12.53, *p* < 0.001; Caspase3+ hair cells: *F*_(2, 101)_ = 4.549, *p* = 0.0128]. Bars are mean ± SD. *n* = 24 neuromasts in control, *n* = 24 in 10 μM SP600125-treated neuromasts, and *n* = 56 in 15 μM SP600125-treated neuromasts. ^*^p < 0.05, significant when compared to control larvae; ^**^p < 0.01, highly significant when compared to control larvae; ^***^p < 0.001, highly significant when compared to control larvae. **(D)** After treatment of 3 dpf larvae with 15 μM SP600125 for 2 days, protein extracts were prepared and subjected to western blot assay using an antibody against cleaved caspase-3. β-Actin was included as the loading control.

After observing the proliferation defect in treated zebrafish larvae, we performed TUNEL analysis on 5 dpf control and SP600125-treated larvae to investigate the roles of JNK in apoptosis. SP600125-treated larvae had significantly greater numbers of TUNEL-positive cells throughout the brain and trunk regions when compared to untreated controls (Supplemental Figures 5C,D). To assess the relative levels of apoptosis in neuromasts, we labeled the zebrafish larvae with anti-cleaved caspase-3 antibody. Very few cleaved caspase-3-positive cells were observed in neuromasts of untreated groups, while the numbers of cleaved caspase-3-positive cells at 5 dpf in the 15 μM SP600125-treated larval neuromasts were significantly increased (Figures 3A–C; *p* < 0.001). Because the *Brn3c:*mGFP transgenic zebrafish lateral line hair cells were labeled with GFP, we were able to determine if the hair cells were cleaved caspase-3-positive. As shown in Figure 3A, we occasionally detected cleaved caspase-3-positive hair cells in control neuromasts. On the contrary, the emergence of cleaved caspase-3-positive hair cells became frequent in larvae at the higher concentrations of SP600125 (15 μM) at 5 dpf (Figures 3B,C; *p* < 0.01). This was further confirmed by the western blot analysis of proteins from zebrafish larvae (Figure 3D).

**Figure 4:**
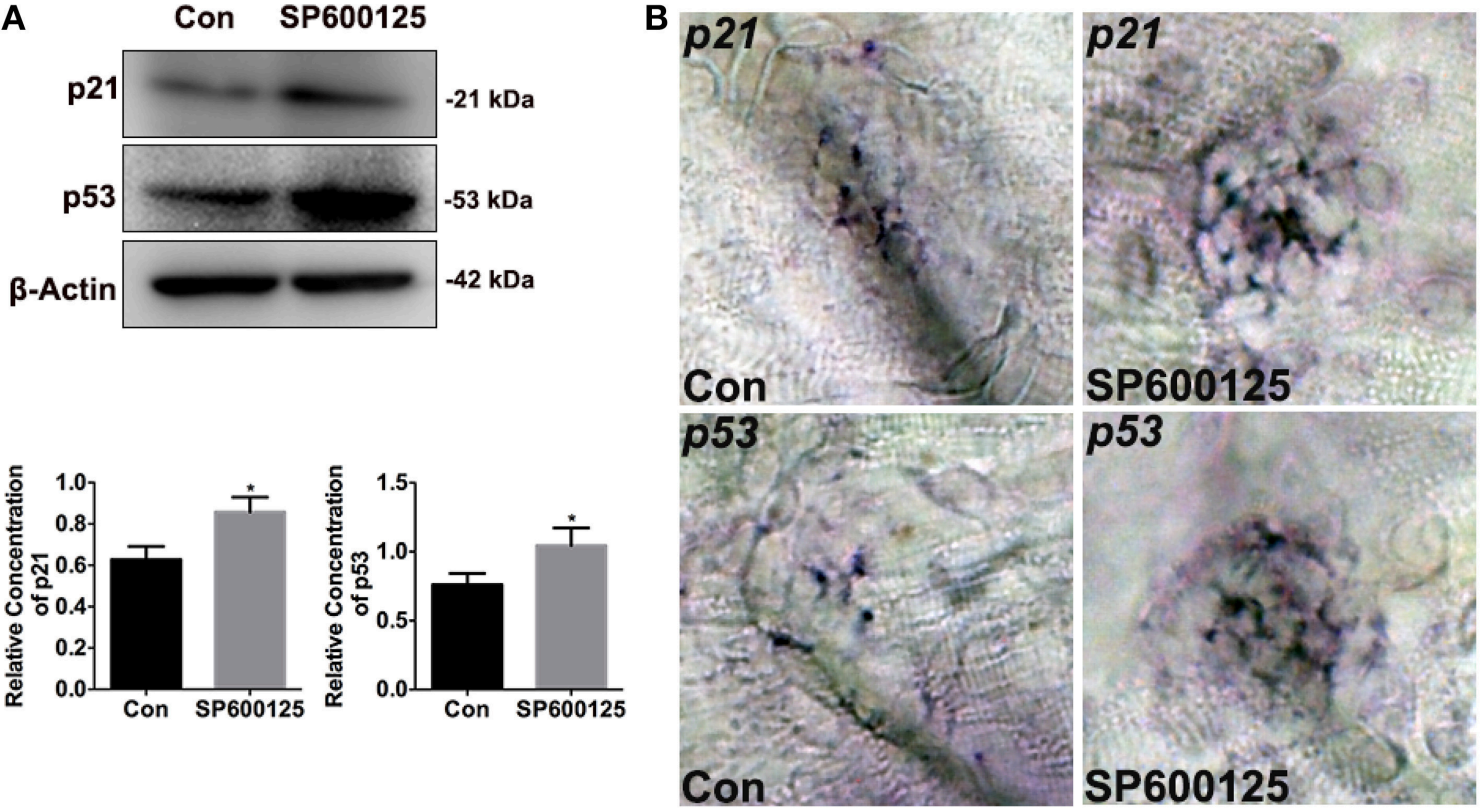
**Effects of SP600125 on the expression of p21 and p53**. **(A)** After treatment of larvae with 10 μM SP600125 for 2 days, protein extracts were prepared and subjected to western blot assay using antibodies against p21 and p53. β-Actin was included as the control. (p21, unpaired *t*-test, two-tailed, *t* = 4.172, *df* = 4, *p* = 0.014; p53, unpaired *t*-test, two-tailed, *t* = 3.273, *df* = 4, *p* = 0.0307).Bars are mean ± SD for three experimental replicates. ^*^p < 0.05. **(B)** Localization of *p21* and *p53* genes with whole-mount *in situ* hybridization in SP600125-treated and untreated larvae.

Previous reports have showed that JNK inhibition induces cell cycle arrest through induction of *p21* expression (Du et al., 2004; Moon et al., 2011). In this study, western blot and whole-mount *in situ* analysis were conducted to determine if SP600125 alters the expression of cell cycle-regulated genes in zebrafish. As shown in Figure 4, exposure to 10 μM SP600125 increased the levels of *p21*. We also examined the expression of *p53* by western blotting and *in situ* analysis, and the expression of *p53* was significantly increased in the treated group (Figure 4). These observations provide evidence that JNK inhibition with SP600125 in zebrafish lateral line neuromast cells induces both cell cycle arrest and apoptosis.

## Discussion

JNK signaling is essential for a wide range of physiological processes and disease states. JNK is generally activated by stress stimuli such as cytokines, growth factors, or cellular damage (Davis, 2000; Johnson and Nakamura, 2007), and activated JNK phosphorylates transcription factors of the Jun family that are involved in the regulation of inflammation, cell proliferation, cell differentiation, apoptosis, and tumorigenesis (Kang et al., 2003; Huh et al., 2004). The biologic outcome of JNK activation is multifaceted and depends on the cell type, stimulus, and the duration of JNK activation (Liu and Lin, 2005). In immortalized neural stem cells, JNK blockade has been shown to inhibit proliferation and to induce increases in p53, p21^Cip1∕Waf1^, and BAX protein levels (Yang et al., 2005). JNK activation contributes to IL-3–mediated cell survival through phosphorylation and inactivation of the proapoptotic Bcl-2 family protein BAD (Yu et al., 2004). Moreover, it was reported that JNK2 blockade in fibroblasts inhibits cell proliferation by promoting G_2_/M-phase arrest and apoptosis (Du et al., 2004). Furthermore, it was shown that JNK signaling is involved in tissue morphogenesis and regeneration (Oliva et al., 2006; Barnat et al., 2010; Hirai et al., 2011). For example, previous studies have shown that JNK activity is essential in axon formation (Oliva et al., 2006; Hirai et al., 2011). Gene targeting studies in mice have demonstrated that inhibition of JNK1 significantly disrupts anterior commissure tract formation, indicating that JNK1 is required for commissural axon guidance in the developing nervous system (Chang et al., 2003). In addition, recent studies showing alterations in JNK signaling in various kinds of tumors indicate that modulation of JNK signaling might be a promising tool for preventing cancer development (Jørgensen et al., 2006; López-Sánchez et al., 2007; Nakamura and Takekawa, 2012; Davies and Tournier, 2012). Remarkably, many studies have shown that the JNK signaling pathway plays a crucial role in hearing loss due to acoustic trauma or aminoglycoside antibiotic (Pirvola et al., 2000; Wang et al., 2003, 2007; Zine and van de Water, 2004; Eshraghi et al., 2007). However, the potential role of JNK in inner ear development is less well-characterized. Given the similarities between zebrafish lateral line hair cells and mammalian inner ear sensory hair cells, the zebrafish model has been used to investigate the activity of JNK in hair cells. Because SP600125 is commonly used for assessing the complex roles of JNK in mediating biological processes (Bennett et al., 2001), we used SP600125 to suppress JNK activity and to evaluate the effect of JNK inhibition on lateral line hair cell development. Our results show that treatment of developing zebrafish with SP600125 led to a dose-dependent decrease in hair cells. Additionally, as assayed by BrdU immunohistochemistry, cell proliferation in neuromasts decreased in response to SP600125 treatment. Finally, apoptosis within the SP600125-treated neuromasts, as measured by cleaved caspase-3 labeling, increased. Thus, our results provide compelling evidence that JNK signaling is involved in the development of zebrafish neuromast hair cells.

JNK1 and JNK2 are expressed in a variety of tissues during development, whereas JNK3 is primarily expressed in the brain, heart, and testes (Gupta et al., 1996; Kuan et al., 1999). Studies of JNK gene deletions have provided more insight into the roles of different JNK isoforms in distinct cellular processes and morphogenesis. For example, knockdown of *jnk1* by RNA interference causes axonal commissure defects and decreased microtubule polymer length (Chang et al., 2003) as well as disturbed dendritic architecture in the brain (Bjorkblom et al., 2005), and this suggests that JNK1 is required for maintaining cytoskeletal integrity in the developing nervous system. Similarly, it was found that deletion of either *jnk2* or *jnk3*, like *jnk1*, was not embryonic lethal, but mice lacking both *jnk1* and *jnk2* die during mid-gestation owing to regional and developmental stage-specific alterations in apoptosis in the developing brain (Kuan et al., 1999; Sabapathy et al., 1999). JNK signaling is best known for its essential role in cell apoptosis following stress, but recent studies also support a role for JNK as an important mediator of normal brain morphogenesis during development (Waetzig et al., 2006), including neural tube closure (Kuan et al., 1999), neurite outgrowth (Oliva et al., 2006; Dajas-Ballador et al., 2008), neuronal migration (Hirai et al., 2002), and lens development in the eye (Weston et al., 2003). There is growing evidence for the molecular mechanisms of JNK action, and JNKs might influence the microtubule cytoskeleton via phosphorylation of the microtubule-stabilizing protein doublecortin (Gdalyahu et al., 2004), the stathmin family of proteins (Tararuk et al., 2006), and MAP2 and MAP1B (Chang et al., 2003).

Previous studies have shown that all three JNK isoforms are expressed in adult dorsal root ganglia neurons and that JNKs are rapidly activated in response to peripheral nerve injury. This activation of JNK in turn activates the transcription factors c-Jun and activating transcription factor-3 (ATF-3). However, JNK-specific inhibition does not affect neuronal survival, but instead dramatically reduces neuritogenesis, c-Jun activation, and ATF-3 induction suggesting that JNK-mediated c-Jun activation and subsequent ATF-3 induction are necessary for promoting axonal outgrowth of sensory neurons in rat dorsal root ganglia (Kenney and Kocsis, 1998; Lindwall et al., 2004; Cavalli et al., 2005). The role of JNKs in the regulation of sensory neurons prompted us to investigate the functions of JNK signaling during hair cell development. Our present findings demonstrate that SP600125 treatment reduces hair cell numbers. Because the proper regulation of cell proliferation and cell death is required for hair cell formation, our second goal in this study is to assay cell proliferation and apoptosis, and our data suggest that failure to enter S phase, as well as increased cell apoptosis, contribute to fewer hair cells in neuromasts treated with JNK inhibitor.

Recent studies have found that JNK activity is involved in the regulation of cell proliferation and apoptosis (Davis, 2000; Lin, 2003), and the inhibition of the JNK pathway with SP600125 is well known to result in cell cycle arrest, endoreduplication, and apoptosis in various cancer cells (Hideshima et al., 2003; Du et al., 2004; Mingo-Sion et al., 2004). Studies using both SP600125 and antisense approaches have suggested a role for JNK in cell cycle progression and tumor cell growth inhibition. For example, targeted depletion of either JNK1 or JNK2 with antisense oligonucleotides caused cell proliferation inhibition associated with S-phase arrest and p53-independent induction of the cyclin-dependent kinase (Cdk) inhibitor *p21* as well as subsequent decreases in both Cdk1 and Cdk2 kinase activity in cancer cells (Potapova et al., 2000). Our results agree well with previous reports showing that JNK inhibition by SP600125 is strongly correlated with cell proliferation as assessed by BrdU staining in zebrafish neuromasts. Because SP600125 is known to induce *p21*, it will be of interest to investigate whether SP600125-induced cell proliferation inhibition in lateral line neuromasts during the course of hair cell development in zebrafish is influenced by changes in the activities of Cdks or Cdk inhibitors such as *p21*. The functions of JNK in cell cycle progression will provide important directions for future studies regarding how JNK affects hair cell development.

Decreases in cell proliferation in the neuromasts of SP600125-treated fish could also be due to induction of apoptosis, so we performed a cell death analysis by cleaved caspase-3 staining. Our present data clearly show that SP600125 treatment induces significant increases in the number of cleaved caspase-3-positive cells in neuromasts compared to controls, and this demonstrates that JNK inhibition induces cell death in neuromasts mainly through caspase-3 activation. Many cellular regulators have been reported to be involved in the induction of apoptosis, for instance, the tumor suppressor protein p53 plays a critical role in the regulation of cell growth, proliferation, and apoptosis (Bates and Vousden, 1999; Vogelstein et al., 2000; Schmitt et al., 2002). The activation of p53 triggers a cascade of gene expression that leads either to growth arrest at the G_1_/S or G_2_/M transitions of the cell cycle or to apoptosis (Appella and Anderson, 2001). Recent studies have linked JNK to apoptosis in multiple ways. JNK's ability to regulate the apoptotic responses might stem from its ability to regulate p53 function. p53 protein has been shown to be capable of inducing both cell cycle arrest and apoptosis by activating *p21*, one of p53's most important transcriptional targets, or by inducing *PUMA, Noxa, Bax*, or other genes that play crucial roles in apoptosis induction. SP600125 is known to directly induce p53 expression, but it has been shown that G_2_/M arrest by SP600125 treatment functions independently of p53 (Mingo-Sion et al., 2004). Western blotting data clearly show that SP600125 treatment increases the protein levels of p21 and p53, and these results provide evidence that JNK inhibition in lateral line neuromasts inhibits proliferation due to S-phase arrest, which is accompanied by induction of p21, and that it induces apoptosis—accompanied by induction of p53—which leads to elevated levels of caspase 3. However, we cannot rule out the possibility that these proteins might also be affected in other tissues where JNK is expressed because the proteins used for immunoblot analysis were isolated from the whole larvae, not only from the neuromasts. We examined the expression of *p21* and *p53* by whole-mount *in situ* analysis, and the expressions were significantly increased in the treated group. These observations provide evidence that JNK inhibition with SP600125 in zebrafish lateral line neuromast cells induces both cell cycle arrest and apoptosis.

In conclusion, our study supports a novel role for JNK in hair cell development in the zebrafish lateral line, and this appears to be through the regulation of cell proliferation and apoptosis in the neuromasts accompanied by induction of *p53* and*p21*. The present study provides new insights into the mechanisms of lateral line hair cell development.

## Author contributions

YH conceived and designed the work. CC and JL performed the zebrafish experiments and data analyses. SS performed the zebrafish experiments and wrote the manuscript. All authors discussed the data. All authors reviewed the manuscript.

### Conflict of interest statement

The authors declare that the research was conducted in the absence of any commercial or financial relationships that could be construed as a potential conflict of interest.

## Supplementary material

Supplementary Figure 1 **Pattern of JNK phosphorylation during hair cell development. (A)** Expression pattern of jnk1 in the neuromast of zebrafish at 5 dpf. **(B)** Extracts from control larvae and SP600125-treated larvae at 5 dpf were run on SDS-PAGE and western blotted with the anti-phosphorylated JNK antibodies. JNK phosphorylation was detected in the control larvae at 5 dpf, but p-JNK was obviously down-regulated after SP600125 treatment.

Supplementary Figure 2 **Effect of JNK inhibition on the development of zebrafish larvae**. Full-body images of 5 dpf zebrafish larvae exposed to 0μM (control) **(A)**, 5μM **(B)**, 10μM **(C)**, and 15μM SP600125 **(D)** during the 3–5 dpf period of hair cell development. The zebrafish larvae treated with 5μM SP600125 from 3 to 5 dpf were morphologically normal when compared to control larvae while more defects were observed in the 15μM-treated larvae, such as pericardium edema and reduced total length.

Supplementary Figure 3 **The number of GFP+ hair cells is decreased in embryos treated with SP600125 for 2 days**. Histograms show the quantitative measurements of the number of hair cells in larvae treated with SP600125. The experiment was repeated three times with consistent results [experiment 1, experiment 2, and experiment 3; One-way ANOVA; experiment 1: *F*(3, 112) = 115.6, *p* < 0.001; experiment 2: *F*(3, 108) = 73.05, *p* < 0.001; experiment 3: *F*(3, 112) = 237.5, *p* < 0.001]. Bars are mean ± SD. *n* = 20–36 neuromasts per treatment. ^***^*p* < 0.001, highly significant difference when compared to control larvae.

Supplementary Figure 4 **Effects of varying duration of SP600125 exposure on hair cell number during the period of embryonic development. (A)** Control group; **(B)** larvae at 3 dpf were treated with 10μM SP600125 for 4 days; (C) larvae at 3 dpf were treated with 10μM SP600125 for 2 days, after which the inhibitor was washed out and hair cells were analyzed after another 2 days. (D) Quantification of FM1-43FX+ hair cells in the neuromast (NM) for each experimental condition [One-way ANOVA; *F*(2, 117) = 234.9, *p* < 0.001]. Bars are mean ± SD. *n* = 36-44 neuromasts per treatment. ^***^*p* < 0.001.

Supplementary Figure 5 **Effects of JNK inhibition on proliferation and apoptosis in the entire zebrafish**. Detection of cell proliferation (A,B) and apoptosis **(C,D)** in the entire zebrafish (5 dpf) exposed to 0μM (control) **(A,C)**, or 15μM SP600125 **(B,D)**.
