# Supplementary figures and images for: JNK Inhibition Inhibits Lateral Line Neuromast Hair Cell Development
Source: Front Cell Neurosci. 2016 Feb 5;10:19. doi: 10.3389/fncel.2016.00019 (PMC4742541; doi:10.3389/fncel.2016.00019)

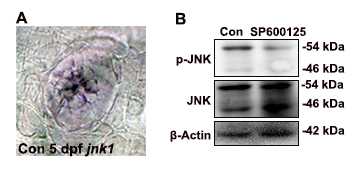

Supplement: Supplementary Figure 1 — Pattern of JNK phosphorylation during hair cell development. (A) Expression pattern of jnk1 in the neuromast of zebrafish at 5 dpf. (B) Extracts from control larvae and SP600125-treated larvae at 5 dpf were run on SDS-PAGE and western blotted with the anti-phosphorylated JNK antibodies. JNK phosphorylation was detected in the control larvae at 5 dpf, but p-JNK was obviously down-regulated after SP600125 treatment. [file Image1.JPEG]

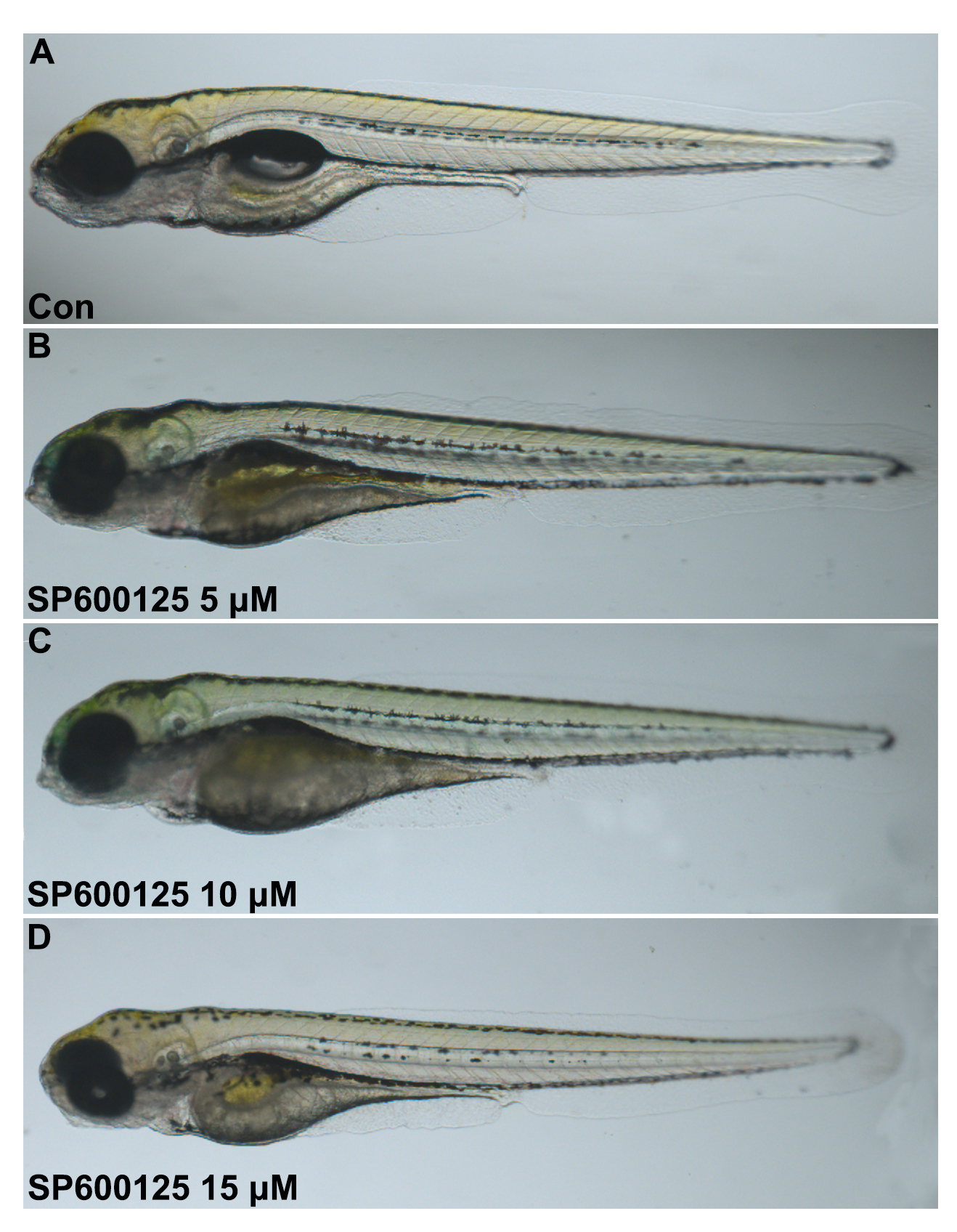

Supplement: Supplementary Figure 2 — Effect of JNK inhibition on the development of zebrafish larvae. Full-body images of 5 dpf zebrafish larvae exposed to 0μM (control) (A), 5μM (B), 10μM (C), and 15μM SP600125 (D) during the 3–5 dpf period of hair cell development. The zebrafish larvae treated with 5μM SP600125 from 3 to 5 dpf were morphologically normal when compared to control larvae while more defects were observed in the 15μM-treated larvae, such as pericardium edema and reduced total length. [file Image2.JPEG]

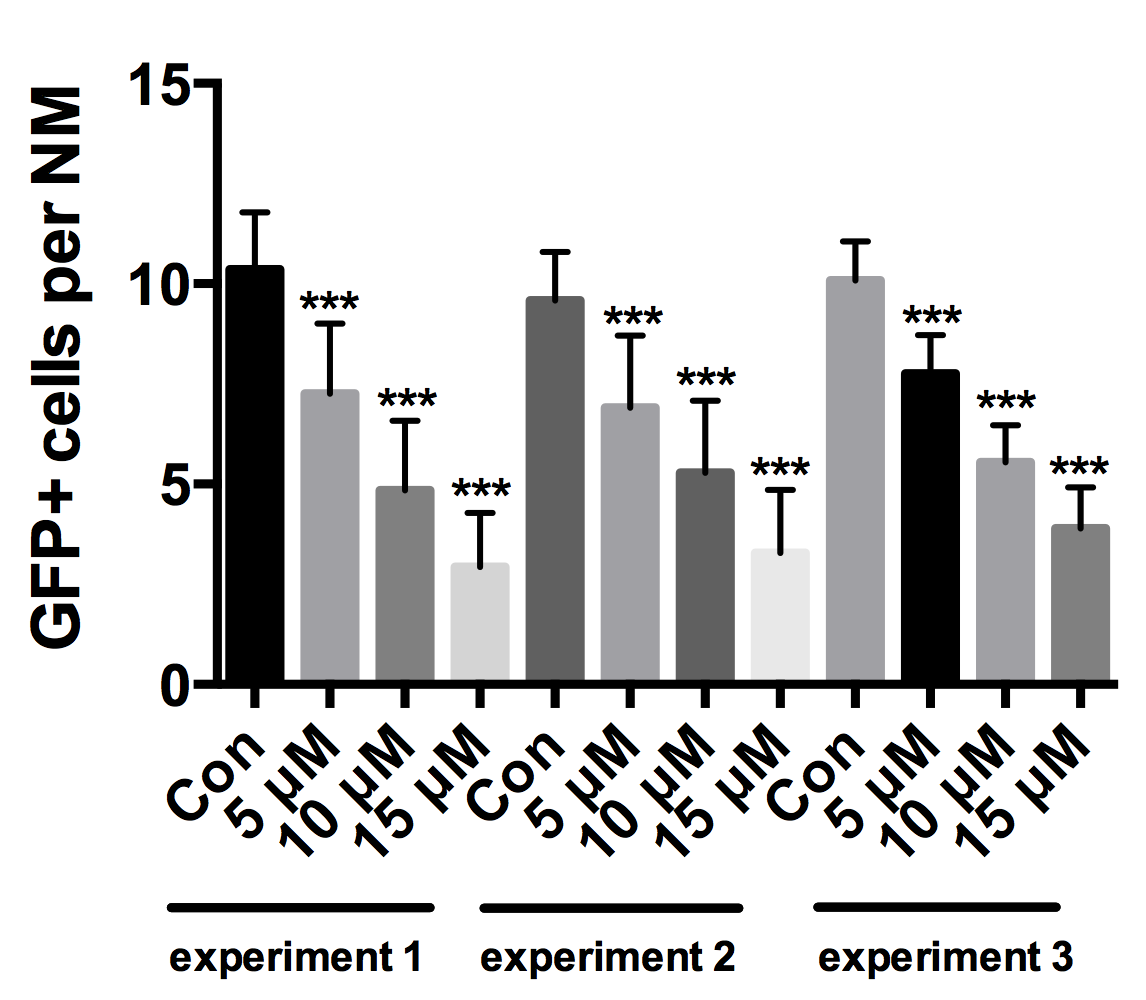

Supplement: Supplementary Figure 3 — The number of GFP+ hair cells is decreased in embryos treated with SP600125 for 2 days. Histograms show the quantitative measurements of the number of hair cells in larvae treated with SP600125. The experiment was repeated three times with consistent results [experiment 1, experiment 2, and experiment 3; One-way ANOVA; experiment 1: F(3, 112) = 115.6, p < 0.001; experiment 2: F(3, 108) = 73.05, p < 0.001; experiment 3: F(3, 112) = 237.5, p < 0.001]. Bars are mean ± SD. n = 20–36 neuromasts per treatment. ***p < 0.001, highly significant difference when compared to control larvae. [file Image3.TIFF]

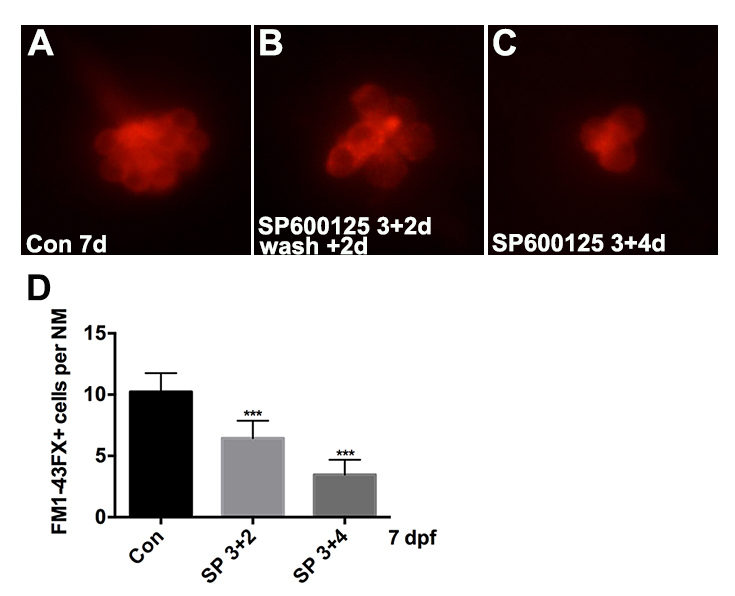

Supplement: Supplementary Figure 4 — Effects of varying duration of SP600125 exposure on hair cell number during the period of embryonic development. (A) Control group; (B) larvae at 3 dpf were treated with 10μM SP600125 for 4 days; (C) larvae at 3 dpf were treated with 10μM SP600125 for 2 days, after which the inhibitor was washed out and hair cells were analyzed after another 2 days. (D) Quantification of FM1-43FX+ hair cells in the neuromast (NM) for each experimental condition [One-way ANOVA; F(2, 117) = 234.9, p < 0.001]. Bars are mean ± SD. n = 36-44 neuromasts per treatment. ***p < 0.001. [file Image4.JPEG]

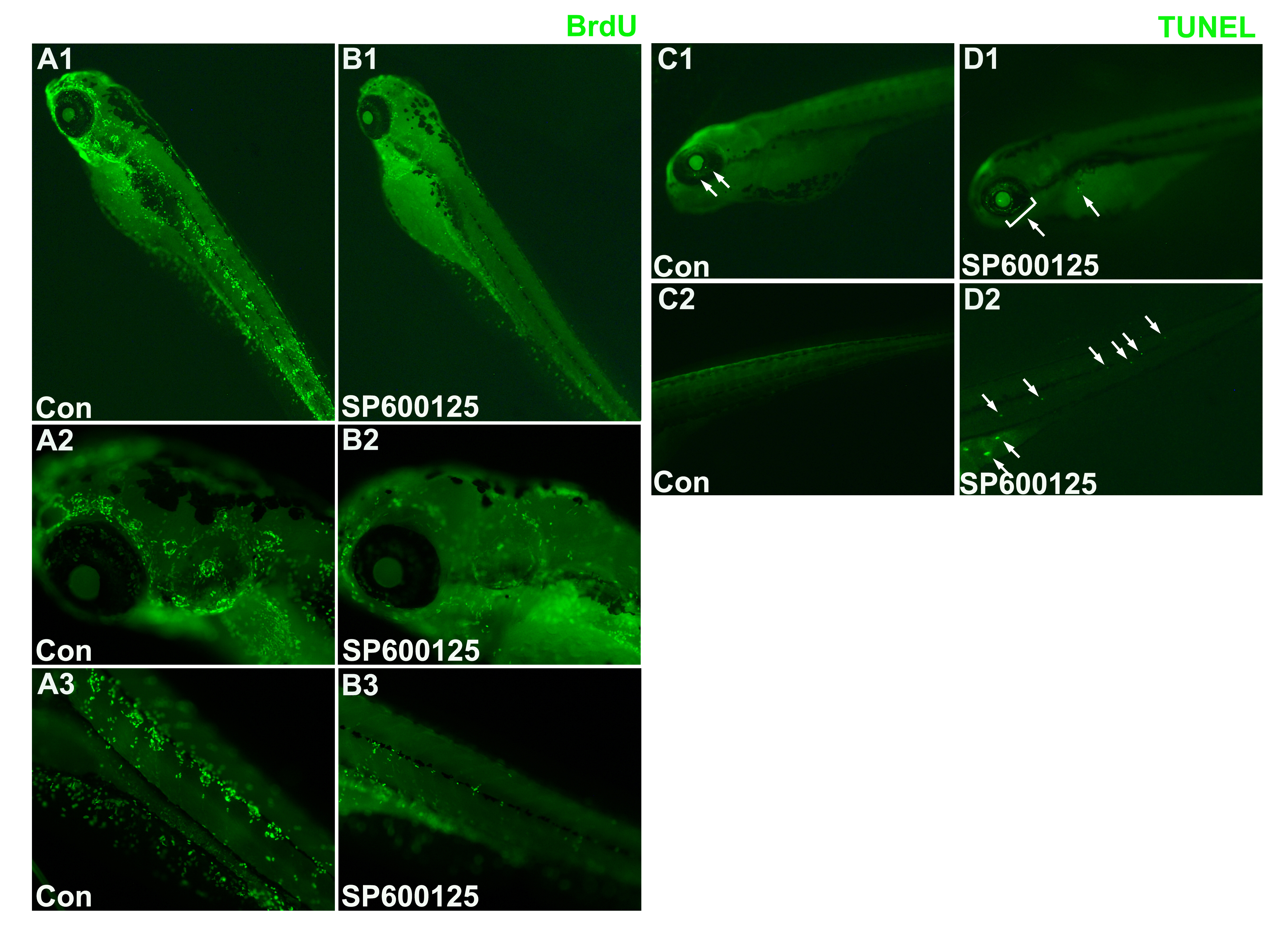

Supplement: Supplementary Figure 5 — Effects of JNK inhibition on proliferation and apoptosis in the entire zebrafish. Detection of cell proliferation (A,B) and apoptosis (C,D) in the entire zebrafish (5 dpf) exposed to 0μM (control) (A,C), or 15μM SP600125 (B,D). [file Image5.JPEG]
